# Bacterial biota of women with bacterial vaginosis treated with lactoferrin: an open prospective randomized trial

**DOI:** 10.1080/16512235.2017.1357417

**Published:** 2017-01-01

**Authors:** Alessandra Pino, Giuliana Giunta, Cinzia L. Randazzo, Salvatore Caruso, Cinzia Caggia, Antonio Cianci

**Affiliations:** ^a^ Department of Agriculture, Food and Environment (Di3A), University of Catania, Catania, Italy; ^b^ Department of General Surgery and Medical Surgical Specialties, Gynecological Clinic, University of Catania, Policlinico Universitario, Catania, Italy

**Keywords:** Vaginal microbiota, Ion Torrent, 16S rDNA, vaginal infection, lactoferrin, *in vivo* trial, *Lactobacillus* spp., *L. helveticus*

## Abstract

**Background:** Bacterial vaginosis is the most frequent condition associated to the vaginal microbiota imbalance, affecting about the 40–50% of women in the world. Even if antibiotics are effcetive for bacterial vaginosis treatment a long-term recurrence rates, higher than 70%, is recorded. Lactoferrin is an iron-binding glycoprotein with bacteriostatic and bactericidal properties. It owns the ability to protect the host against infection, by binding and regulating the iron needed for the bacterial proliferation.

**Objective**: The present study was an open prospective randomized trial (registration no. SHI-EVE-2014.01) aimed at characterizing the bacterial biota of women affected by bacterial vaginosis (BV) and assessing the effects of two different lactoferrin concentrations (100 mg and 200 mg vaginal pessaries) on the composition and dynamics of the vaginal bacterial biota.

**Design**: Sixty women with BV were recruited and randomized into two groups to receive lactoferrin pessaries for 10 days. Clinical evaluation was based on Amsel criteria and Nugent scores. Culture-dependent methods and Ion Torrent PGM sequencing of the 16S rRNA gene were applied to study in depth the overall structure of the vaginal bacterial biota and its dynamics during the treatment.

**Results**: Vaginal lactoferrin administration modified the vaginal microbiota composition in patients with BV. During treatment, both 100 mg and 200 mg lactoferrin vaginal pessaries significantly decreased the occurrence of bacteria associated with BV, such as *Gardnerella*, *Prevotella*, and *Lachnospira*, and increased the occurrence *of Lactobacillus* species. The bacterial biota balance was maintained up to 2 weeks after treatment only in women treated with 200 mg lactoferrin pessaries.

**Conclusions**: This study indicates that lactoferrin could be proposed as an alternative therapeutic approach for BV. Our data showed, for the first time, the dominance of *Lactobacillus helveticus* species during and after vaginal lactoferrin treatment.

## Introduction

The human vaginal microbiota is a complex ecosystem that plays an important role in women’s health, having a balanced mutualistic association with the vaginal environment. In this relationship, the host provides benefit to the microbial communities in the form of nutrients needed to support bacterial growth, and the vaginal microbiota plays a protective role in preventing colonization by potentially pathogenic organisms [1]. Several studies have shown that a healthy vaginal environment is dominated by lactobacilli [2], mainly *Lactobacillus crispatus*, *L*. *gasseri*, *L*. *jensenii*, and *L*. *iners*, followed by *L*. *acidophilus*, *L*. *fermentum*, *L*. *plantarum*, *L*. *brevis*, *L*. *casei*, *L*. *vaginalis*, *L*. *delbrueckii*, *L*. *salivarius*, *L*. *reuteri*, and *L*. *rhamnosus* species [3,4]. These microorganisms, which represent 80–95% of the resident bacteria, play a protective role against pathogens by different mechanisms such as the production of lactic acid, resulting in a low pH (3.5–4.5) [5,6]; the enhancement on the host’s innate immune system [7]; and the production of antimicrobial compounds, including target-specific bacteriocins [8,9] and the broad-spectrum hydrogen peroxide (H_2_O_2_) [6,10]. The most frequent condition associated with imbalance in the vaginal microbiota is bacterial vaginosis (BV), which affects about 40–50% of women in the world [11–13]. The etiology of BV is unclear, but it is currently considered a polymicrobial disorder in which lactobacilli are reduced, absent, or lacking specific antimicrobial properties (i.e. production of H_2_O_2_) with a concurrent increase in opportunistic pathogenic bacteria, mainly *Gardnerella vaginalis* but also anaerobes such as *Atopobium vaginae*, *Bacteroides, Mobiluncus*, *Prevotella, Peptostreptococcus* spp., *Ureaplasma urealyticum*, and *Mycoplasma hominis* [1,14,15]. Treatment with antibiotics, such as metronidazole and clindamycin, is indicated in both symptomatic and asymptomatic women affected by BV [15,16]. Even if antibiotic medication is effective in treating BV, about 25% of women will develop a new episode of BV within 4 weeks [17,18] and the long-term recurrence rates are > 70% [15,19]. Moreover, there are several disadvantages associated with antibiotic therapies, including superinfections with pathogenic microorganisms, susceptibility of lactobacilli to clindamycin, and an increased drug resistance by vaginal pathogens, particularly *G. vaginalis* and anaerobic bacteria [15,20–22]. For these reasons, alternative therapeutic agents need to be sought.

Lactoferrin is an approximately 80 kDa iron-binding glycoprotein belonging to the transferrin family, which is produced and stored in specific (secondary) neutrophil granules and released during neutrophil activation and degranulation. Lactoferrin has bacteriostatic and bactericidal properties, with the ability to protect the host against infection, by binding and regulating the iron needed for bacterial proliferation [23–25]. The antimicrobial effect of lactoferrin is also due to immunomodulation and cytoplasmic membrane disruption of the target cell. Lactoferrin has an inhibitory effect on lipopolysaccharide-induced production of inflammatory cytokines [tumor necrosis factor-α, interleukin (IL)-1β, IL-6, and IL-8 messenger RNA] and interferes with nuclear factor-κB activation in monocytic cells [26,27]. Additional functions of lactoferrin have been reported, such as neutrophil and macrophage activation [28], regulation of specialization and function of lymphocytes [29], activation of natural killer cells [30], control of oxidation injury [29], and down-modulation of ongoing immune-inflammatory responses during preterm delivery [31]. Moreover, lactoferrin exhibits a synergistic effect with immunoglobulin A, lysozyme, antibiotics, and drugs, which helps in the eradication of microorganisms [32]. Giunta and co-workers [31] demonstrated that oral administration of lactoferrin 100 mg, twice a day for 1 month, led to the reduction of IL-6 in cervicovaginal fluid and the normalization of vaginal microbiota in women at preterm delivery risk. Otsuki and Imai [33] showed that, in women with a history of multiple pregnancy losses or preterm delivery and refractory BV, lactoferrin administration could improve the vaginal microbiota, preventing both cervical inflammation and preterm delivery. Sessa et al. [34] reported that intravaginal administration of lactoferrin resolved asymptomatic *Chlamydia trachomatis* in six out of seven pregnant women and resulted in normal deliveries.

The aims of the present study were: (i) to characterize the bacterial biota in women affected by BV and (ii) to assess the effect of two different concentrations of vaginally administered lactoferrin (100 mg and 200 mg) on the vaginal bacterial biota. For this purpose, the vaginal microbiota composition and dynamics of BV-positive women were investigated before, during, and after lactoferrin administration, using an integrated approach based on culture-dependent methods and Ion Torrent™ PGM™ sequencing of 16S ribosomal RNA (rRNA) gene-based amplicons.

## Materials and methods

### Study design and patient enrollment

The present study was an open prospective randomized trial with two parallel groups. From October 2015 to May 2016, 60 BV-positive women were recruited at the Department of General Surgery and Medical Surgical Specialties, Gynecological Clinic, University of Catania (Catania, Italy). Participants were randomly (1:1 ratio) allocated to one of the two experimental groups, A or B, and treated for 10 days with 200 mg or 100 mg lactoferrin, respectively. Vaginal lactoferrin pessaries were kindly provided by AG Pharma s.r.l. (Rome, Italy). Randomization was carried out using a random number generator. Lactoferrin was administered as vaginal tablets and each subject was instructed to apply the tablet once a day, preferably at night before going to bed. All the women underwent a complete assessment in three scheduled appointments: at baseline (T0), on the 10th day of treatment (treatment: T1), and 2 weeks after the end of lactoferrin administration (post-treatment: T2). Inclusion criteria were sexually active women of reproductive age (18–45 years old) with regular menstrual cycles and with symptomatic acute BV diagnosed according to Amsel’s criteria [33] and Nugent score [34]. The exclusion criteria were age < 18 years; Nugent score < 7; known active infection due to *Chlamydia*, yeasts, *Neisseria gonorrhoeae*, or *Trichomonas vaginalis*; clinically apparent herpes simplex infection or defined diagnosed human papillomavirus, herpes simplex virus type 2, or human immunodeficiency virus type 1 infection; pregnancy or breastfeeding; antibiotic, probiotic, or exogenous hormone treatments; and other gynecological conditions that could cause bleeding (polyps, endometrial hyperplasia, etc.). Sexually transmitted infections caused by organisms such as *Chlamydia trachomatis*, *N. gonorrhoeae*, and *T*. *vaginalis* were excluded from the study, so that targeted antibiotic therapy could be administered. Participants were not allowed to use probiotics, even if taken orally, or antibiotics 2 months before recruitment or at any time during the study. Demographics and medical history concerning contraceptive use, infectious disease history, sexual activity, and last menstrual period were assessed at baseline. Pelvic examination, assessment of clinical signs and symptoms of vaginosis, and vaginal discharge sampling were performed at each intervention point (T0, T1, and T2).

The study was conducted in accordance with the Helsinki Declaration (2000) of the World Medical Association and current standards of good clinical practice, and informed written consent was obtained from all participants before enrollment. The study protocol was approved by the local ethics committee (registration number SHI-EVE-2014.01).

### Sample collection

Vaginal discharge samples were obtained from the lateral vaginal wall and the posterior vaginal fornix using sterile cotton-tipped swabs. For each participant, four vaginal swabs were collected at baseline (T0), during the treatment (T1), and post-treatment (T2). In detail, two vaginal swabs were used to assess the BV status: the first one was used for microscopic examination of the fresh smear (detection of clue cells and Gram staining) and the second one for the whiff-amine test. In addition, two swabs, filled with transport medium, were collected and used for microbiological counts and DNA isolation. Vaginal samples were collected at the Department of General Surgery and Medical Surgical Specialties, Gynecological Clinic, University of Catania (Catania, Italy), and immediately transferred, under refrigerated conditions, to the Laboratory of Microbiology of the Department of Agriculture, Food and Environment, University of Catania (Catania, Italy). Vaginal fluid pH was measured during each visit using pH test strips (McKesson, San Francisco, CA, USA).

### Evaluation of clinical criteria

Three out of four Amsel criteria were necessary for the clinical diagnosis of BV: (i) homogeneous, thin, grayish-white vaginal discharge; (ii) vaginal pH > 4.5; (iii) positive whiff-amine test; and (iv) clue cells present on a wet mount of vaginal fluid [35]. The vaginal discharge was subjected to Nugent score determination and to whiff-amine test on two different glass slides. The Nugent score was assessed on a 10-point scale, performing a Gram stain followed by optical microscopic observation under oil immersion. Large Gram-positive bacilli were assumed to be the *Lactobacillus* morphotype, smaller Gram-variable bacilli were assumed to be the *Gardnerella* morphotype, and other organisms were categorized by morphology only, e.g. Gram-negative bacilli, curved rods, Gram-positive cocci in chains, and fusiform. A score of 0–3 was interpreted as *Lactobacillus-*predominant normal vaginal microbiota, a score of 4–6 was considered as intermediate, and a score of 7–10 was assumed as BV-like conditions, with the dominance of small Gram-negative and Gram-variable straight and curved rods. Microscopic observation of vaginal glass smears was also performed to detect the presence of ‘clue cells’. Moreover, according to Amsel’s criteria, the whiff-amine test was carried out. In detail, the presence of a ‘fish odor’, attributable to the production of volatile amines, was evaluated by adding a drop of 10% KOH directly to the glass surface, and the presence of fish odor was detected by the researcher’s sense of smell. The Amsel’s criteria and Nugent score evaluation were also performed at the T1 and T2 sampling times.

### Microbiological analysis

Vaginal discharge, collected using a sterile synthetic-swab tip Transystem Amies Medium Clear (Biolife Srl, Milan, Italy), was analyzed as follows. After dislodging the cells in sterile phosphate-buffered saline (PBS), serial dilutions were made and plated on the following agar media and conditions: Rogosa SL agar (Biolife) incubated at 35–37°C for 40–48 h for *Lactobacillus* counts; Streptococcus Selective Agar (Biolife) incubated at 32°C for 24 h for streptococci; *Gardnerella vaginalis* Selective Medium (Oxoid, Milan, Italy) incubated at 37°C for 40–48 h for *G*. *vaginalis*; MacConkey Agar Mug (Biolife) incubated at 37°C for 16–18 h for *Escherichia coli*; Mannitol Salt Agar (Oxoid) incubated at 32°C for 48 h for staphylococci; Slanetz Bartley Agar (Biolife) incubated at 37°C for 48 h for enterococci; and Chromogenic Candida Agar (Biolife) incubated at 35–37°C for 48 h for *Candida albicans*, *C. tropicalis*, and *C. krusei*. All analyses were performed in duplicate.

### RNA isolation from vaginal swabs

All vaginal samples underwent RNA isolation using the Stool total RNA purification kit (Norgen, Thorold, ON, Canada). The total RNA was treated with RNase-free DNase I (Roche, Almere, Netherlands; 10 U of DNase/20 μg of RNA) for 20 min at room temperature. The quality and concentration of the RNA extracts were determined using 1% agarose–0.5× TBE gels and spectrophotometric measurements at 260, 280, and 230 nm obtained using a NanoDrop® ND-1000 spectrophotometer. The total RNA extracted was retrotranscribed to complementary DNA (cDNA) using random hexamers and a Tetro cDNA synthesis kit (Bioline, Freiburg, Germany), according to the manufacturer’s instructions. The obtained cDNA was subjected to Ion Torrent 16S rRNA gene-based analysis.

### Ion Torrent 16S rRNA gene-based analysis

cDNA amplification and Ion Torrent PGM sequencing of 16S rRNA gene-based amplicons were performed by GENPROBIO srl (Parma, Italy). cDNA obtained from vaginal swab specimens at times T0, T1, and T2 was amplified using the primer pair Probio_Uni /Probio_Rev, which targets the V3 region of the 16S rRNA gene sequence [36]. DNA was amplified under the polymerase chain reaction (PCR) conditions described previously [37]. PCR amplicons were analyzed by electrophoresis on an Experion workstation (Bio-Rad, UK) and quantified using the Experion system (Bio-Rad, UK). Emulsion PCR was performed using the Ion OneTouch™ 200 Template Kit v2 DL (Life Technologies, San Francisco, CA, USA) according to the manufacturer’s instructions. Sequencing analysis was carried out according to the protocol of the Ion Torrent PGM system and using the Ion Sequencing 200 kit. Sequence reads were analyzed by PGM software to delete low-quality and polyclonal sequences. High-quality sequences were trimmed and filtered with the default settings, using QIIME pipeline version 1.4.0 (http://qiime.sourceforge.net). Filtered sequences were exported as sff files.

### Taxonomic identification

The sff sequence files were processed using QIIME [38]. The sequences were first clustered into operational taxonomic unit (OTU) clusters with 97% identity (3% divergence). All reads were classified to the lowest possible taxonomic rank using QIIME and a reference data set from the Ribosomal Database Project [39]. OTUs were assigned using uclust [40]. Alpha-diversity (rarefaction, Good’s coverage, Chao1, richness, and Shannon diversity indices) and beta-diversity measures were calculated and plotted using QIIME. Final data sets at species and other relevant taxonomic levels were compiled into separate worksheets for compositional analysis among the samples. Differences in microbial communities between vaginal samples were also investigated using the unweighted pair group method with arithmetic mean (UPGMA) clustering on the distance matrix of OTU abundance. This resulted in a Newick formatted tree, which was obtained using the QIIME package [37].

### Statistical analysis

Analysis of variance (ANOVA) was carried out on transformed data followed by separation of means with Tukey’s HSD, using the statistical software Statistica 6.0 for Windows 1998 (StatSoft, Vigonza, Italy).

## Results

### Study population

**Figure 1.**
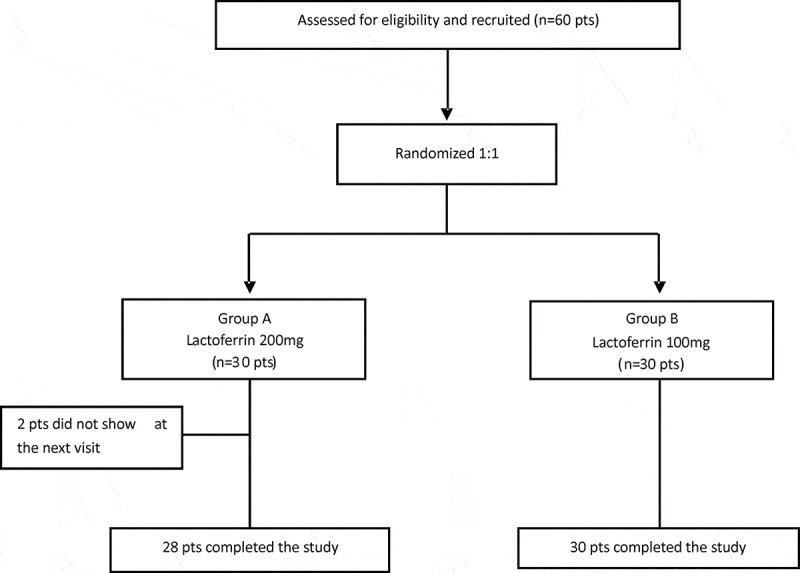
Design of the open prospective randomized trial.

Sixty eligible subjects were prospectively enrolled in the study. At the enrollment visit, the participants were randomly assigned to receive the lactoferrin 200 mg vaginal tablets (group A) or the lactoferrin 100 mg vaginal tablets (group B) for 10 days. The two groups were homogeneous for age, height, and weight distribution. In addition, BV signs and symptoms (itching, burning, dysuria, and odor) were similar in groups A and B (supplementary Table S1). In total, 58 participants completed the study, as there were two dropouts in group A (Figure 1).

### Clinical criteria

As reported in Table 1, at baseline both groups A and B had Nugent scores ≥ 7, confirming the BV status.

**Table 1.** Clinical characteristics of groups A and B at baseline (T0), on the 10th day after the start of lactoferrin supplementation (treatment: T1), and 2 weeks after stopping lactoferrin administration (post-treatment: T2).

		Nugent score				
Group	Time of intervention	≤ 3	4–6	≥ 7	Positive whiff test	Vaginal pH > 4.5
A (*n* = 28) Lactoferrin 200 mg	T0	0	0	28	27	28
	T1	24	2	2	1	5
	T2	21	5	2	2	3
B (*n* = 30) Lactoferrin 100 mg	T0	0	0	30	28	30
	T1	20	5	5	4	8
	T2	13	9	8	7	12

The results, related to patients who completed the study, are expressed as absolute numbers.

In group A, the lactoferrin 200 mg treatment (T1) significantly reduced the Nugent score to below the threshold of 7 in 26 out of 28 participants (92.8%). In detail, two participants showed an intermediate microbiota (Nugent score 4–6) and the other 24 (85.7%) had a score ≤ 3, indicating a restoration of the vaginal ecosystem. Few shifts were detected 2 weeks after stopping lactoferrin 200 mg administration (T2). The majority of participants (26/28) had Nugent scores below the threshold of 7. Moreover, only five and three participants at T1 and T2 sampling times, respectively, had a vaginal pH > 4.5. The whiff-amine test percentage of positivity shifted from 96.4% at baseline (T0) to 3.6% after 10 days of lactoferrin 200 mg administration (T1), and to 7.1% 2 weeks after stopping lactoferrin 200 mg treatment (T2).

In group B, the lactoferrin 100 mg treatment (T1) reduced the Nugent score to below the threshold of 7, in 25 participants (83.3%) (Table 1). In particular, 20 participants (66.6%) had Nugent scores ≤ 3, five (16.7%) had intermediate microbiota (Nugent score 4–6) and the remaining five (16.7%) had a Nugent score ≥ 7. Two weeks after stopping lactoferrin 100 mg administration (T2), only 13 participants (43.3%) had a Nugent score ≤ 3. The remaining 17 participants had intermediate (nine out of 30) or BV (eight out of 30) microbiota. The vaginal pH value was > 4.5 in all participants at baseline (T0) and shifted to physiological values at T1 and T2 sampling times in 22 and 18 participants, respectively. The percentage of positivity on the whiff-amine test was characterized by a pronounced reduction from T0 to T1 and a slight increase after lactoferrin administration (T2) (Table 1).

### Microbial counts

Microbial counts, expressed as the mean and standard deviation of log cfu/ml of the main microbial groups detected during the whole of the study, are reported in Table 2. At baseline (T0), both groups had a complex microbiota dominated by potentially pathogenic bacteria with a low cell density of lactobacilli. In group A, the lactoferrin treatment significantly reduced the cell density of all the microbial groups studied except for enterococci (*p* = 0.398) and *Candida* spp. (*p* = 0.329). As expected, the level of lactobacilli significantly increased (*p* < 0.001). This trend was also observed post-treatment (T2). Comparing baseline (T0) and post-treatment (T2) sampling times, statistically significant change was detected for all the microbial groups investigated (Table 2). In group B, the treatment led to a significant reduction in viable cells of *Gardnerella* and *Enterococcus* spp. At the same sampling time, there was an increase in viable cells of *Lactobacillus* spp. (*p* < 0.001). Two weeks after stopping lactoferrin 100 mg administration (T2), significant changes in viable cells were detected for all the microbial groups investigated, except for *E. coli* and *Candida* (Table 2).

**Table 2.** Microbial counts and significance (ANOVA) of groups A and B during the intervention.

	Microbial count (log cfu/ml)											
	Group A (lactoferrin 200 mg)						Group B (lactoferrin 100 mg)					
Microbial group	T0	T1	T2	*p* T0 vs T1	*p* T0 vs T2	*p* T1 vs T2	T0	T1	T2	*p* T0 vs T1	*p* T0 vs T2	*p* T1 vs T2
*Staphylococcus* spp.	3.42 ± 0.02	2.87 ± 0.04	2.16 ± 0.02	0.030	0.000	0.004	2.16 ± 0.05	1.96 ± 0.05	1.49 ± 0.07	0.165	0.003	0.001
*Gardnerella* spp.	3.37 ± 0.01	2.43 ± 0.02	1.47 ± 0.02	0.000	0.000	0.000	2.44 ± 0.05	1.76 ± 0.04	1.46 ± 0.06	0.000	0.000	0.026
*Streptococcus* spp.	4.59 ± 0.01	4.06 ± 0.01	2.90 ± 0.01	0.015	0.000	0.000	3.41 ± 0.04	3.40 ± 0.05	2.99 ± 0.08	0.465	0.012	0.002
*Enterococcus* spp.	4.50 ± 0.03	4.44 ± 0.02	3.25 ± 0.01	0.398	0.005	0.000	3.52 ± 0.07	3.17 ± 0.05	2.84 ± 0.05	0.004	0.001	0.016
*Escherichia coli*	1.02 ± 0.01	0.61 ± 0.05	0.37 ± 0.00	0.047	0.004	0.002	0.39 ± 0.03	0.41 ± 0.04	0.29 ± 0.03	0.423	0.187	0.084
*Candida* spp.	0.47 ± 0.04	0.43 ± 0.00	0.21 ± 0.00	0.329	0.011	0.053	0.07 ± 0.01	0.02 ± 0.00	0.03 ± 0.00	0.072	0.072	ND
*Lactobacillus* spp.	3.95 ± 0.02	5.54 ± 0.02	5.72 ± 0.02	0.000	0.000	0.181	4.08 ± 0.12	4.86 ± 0.05	4.72 ± 0.10	0.000	0.000	0.205

Data are shown as mean ± SD.

### Ion Torrent 16S rRNA gene-based analysis

From the vaginal swab specimens of groups A and B, collected at T0, T1, and T2 sampling times, PCR amplicons of the V3 hypervariable region of the 16S rRNA gene were subjected to Ion Torrent. Mean values of 63,455 and 59,316 high-quality reads were obtained from groups A and B, respectively. The highest (95,862) and the lowest (41,246) values of reads were obtained from samples collected in group A at T0 and T2, respectively (Table S2). The bacterial community was analyzed using rarefaction curves, a species-level measure (OTU), a richness estimator (Chao1), and a Shannon diversity index (Table S2). Rarefaction analysis provides a powerful method for evaluating the diversity and richness of the vaginal microbiota. For both groups A and B, the rarefaction curves highlighted that there was much more richness in bacterial diversity at baseline (T0) than after 10 days of lactoferrin administration (T1) and 2 weeks after stopping lactoferrin (T2) (supplementary Figure S1). Moreover, the biodiversity observed and the estimated sample coverage (Good’s coverage value) for the V3 hypervariable region of the 16S rRNA gene of the vaginal swab samples indicated that satisfactory coverage (> 99%) of the biodiversity was achieved (Table S2). Overall, the OTU, Chao1, and Shannon index values of vaginal specimens collected from groups A and B decreased during the lactoferrin administration. Two weeks after stopping lactoferrin (T2), all indices continued to decrease in group A and to increase in group B, indicating a higher biodiversity.

**Figure 2.**
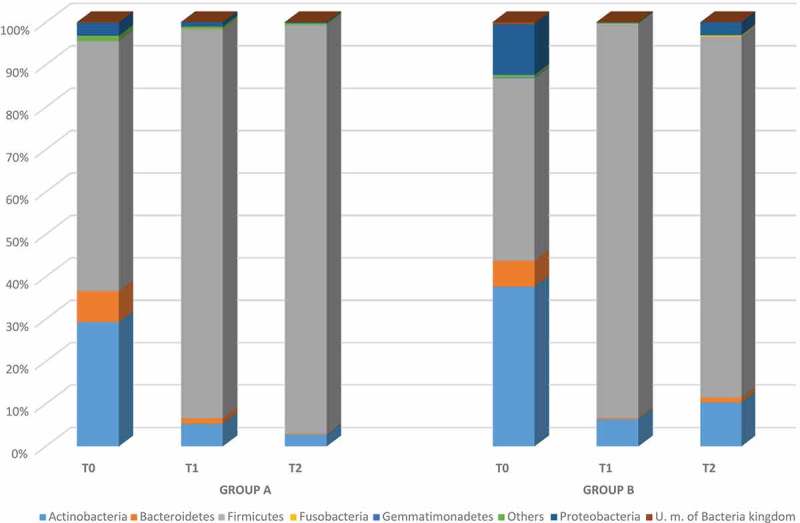
Relative abundance of vaginal bacterial phyla obtained from patients treated with lactoferrin 200 mg (group A) and 100 mg (group B) at baseline (T0), during the treatment (T1), and post-treatment (T2).

**Figure 3.**
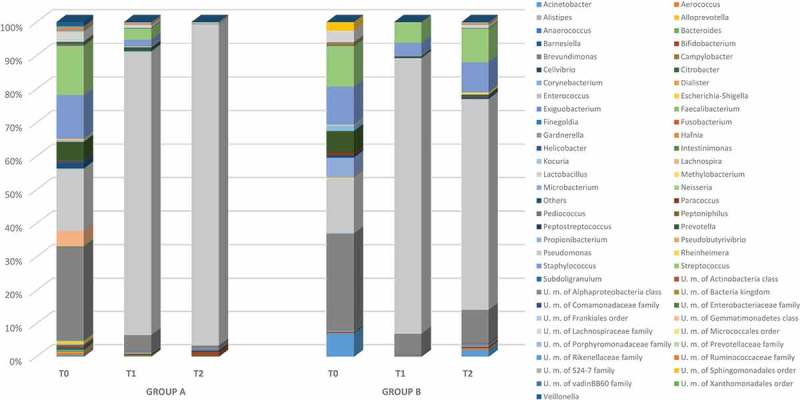
Relative abundance of vaginal bacterial genera obtained from patients treated with lactoferrin 200 mg (group A) and 100 mg (group B) at baseline (T0), during the treatment (T1), and post-treatment (T2).

The relative abundance of the predominant phyla detected in groups A and B is reported in Figure 2. In total, seven phyla were found at baseline and the vast majority belonged to Firmicutes, Actinobacteria, Bacteroidetes, and Proteobacteria, followed by Fusobacteria, Gemmatimonadetes, and others. It should be highlighted that, after 10 days of lactoferrin administration at both 200 mg and 100 mg, all dominant phyla decreased, except for Firmicutes. Differences were achieved between groups A and B 2 weeks after stopping treatment. In group A, all phyla continued to decrease except for Firmicutes, which represented more than 96% of the total microbiota. In contrast, group B was characterized by a significant increase in Actinobacteria (from 6% to 10%) and Proteobacteria (from 0.1% to 3%), followed by a slight increase in Bacteroidetes (from 0.30% to 1%). Firmicutes, despite showing a slight decrease, dominated after stopping treatment (Figure 2). At the genus level (Figure 3), a total of 61 different genera occurred at baseline, with 55 and 32 in groups A and B, respectively. In detail, eight genera (*Gardnerella*, *Lactobacillus, Streptococcus*, *Staphylococcus, Prevotella*, *Lachnospira*, unclassified member of Lachnospiraceae family, and *Veillonella*) constituted more than 87% of the vaginal microbiota of group A and 78% of group B. In addition, *Acinetobacter*, *Microbacterium*, an unclassified member of the order Sphingomonadales, and *Propionibacterium* genera represented 17% of the genera detected in group B.

**Figure 4.**
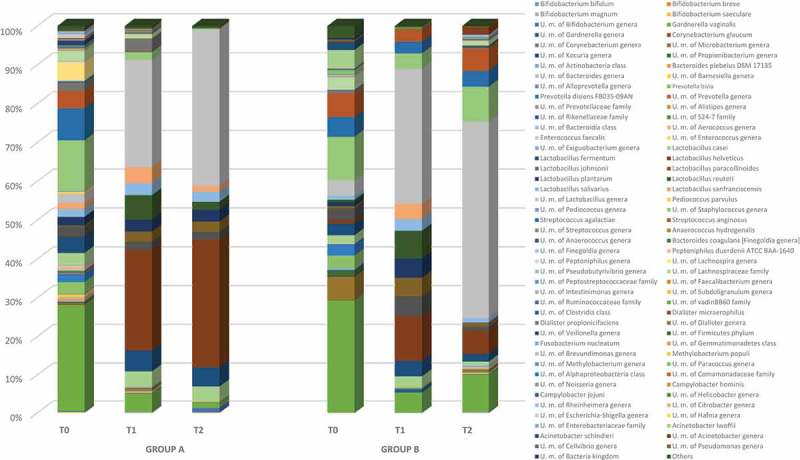
Relative abundance of vaginal bacterial species obtained from patients treated with lactoferrin 200 mg (group A) and 100 mg (group B) at baseline (T0), during the treatment (T1), and post-treatment (T2).

At the species level, overall 90 different species were detected (Figure 4). In detail, at baseline both groups were characterized by the occurrence of *G*. *vaginalis* species (28%), followed by species ascribed to *Lactobacillus* genus (*L. fermentum*, *L. casei*, *L. jonsonii*, and *L. plantarum*) (18%), *Streptococcus* spp. (*S. agalactiae* and *S. anginosus*) (13%), *Staphylococcus* spp. (12%), *Prevotella bivia* (3%), and *Prevotella disiens* (3%) (Figure 4). Species ascribed to the genus *Lactobacillus* increased during the treatment in both groups A and B, whereas *G*. *vaginalis*, *S. agalactiae*, *S. anginosus*, *Staphylococcus* spp., *P*. *bivia*, and *P*. *disiens* significantly decreased until 2 weeks after stopping lactoferrin.

Different species occurrence was registered 2 weeks after stopping lactoferrin treatment, with the exception of *Lactobacillus* spp., which overall increased in both groups. However, within *Lactobacillus*, *L*. *helveticus* increased in group A, reaching 33% of occurrence, while in group B it decreased, registering 6% of occurrence. Similar trends were seen for *L. fermentum*, *L. casei*, *L. jonsonii*, and *L. plantarum* (Figure 4).

## Discussion

BV is the most commonly reported microbiological syndrome affecting millions of women of childbearing age. BV is characterized by a shift in the vaginal microbiome from the dominant *Lactobacillus* to a polymicrobial dysbiosis, which involves multiple bacterial species that may vary from patient to patient [41]. To investigate the vaginal bacterial composition of BV-positive women, 60 patients with suspected vaginal dysbiosis were enrolled in this study after a clinical diagnosis based on Amsel criteria and Nugent scores. The latter is currently considered to be the gold standard for BV diagnosis in research settings [36]. However, recent studies have shown that the use of non-culture-based tools such as quantitative and/or qualitative molecular fingerprinting methods and sequence analysis of the 16S rRNA provide a full understanding of the taxonomic composition of the vaginal microbiota, its community structure, and its function [42]. In the present study, culture-based microbiological criteria were coupled with culture-independent methods for in-depth understanding of the overall structure of the vaginal bacterial biota. The approach based on Ion Torrent PGM sequencing of the 16S rRNA gene is a novel high-throughput method with a faster turnaround than other next-generation sequencing techniques [43,44]. Application of the Ion Torrent technology was suitable for the investigation of the bacterial ecology in the BV vaginal ecosystem, allowing us to detect its compositional change at phylum, genus, and species level. Overall, our results revealed a high level of bacterial diversity in BV-positive women, in accordance with a previous study [42]. Of the seven phyla that we detected in the BV vaginal ecosystem, Firmicutes constituted about 50% of the total bacteria biota, followed by Actinobacteria (34%), Proteobacteria (8%), and Bacteroidetes (7%), mainly associated with BV. Although numerous studies have revealed an association between BV and the presence of *Gardnerella*, *Atopobium*, *Prevotella*, *Bacteroides*, *Peptostreptococcus*, *Mycoplasma*, and others [45,46], the role of these bacteria in the etiology and pathology of the dysbiosis remains unclear. In addition, from historical studies of BV, no single bacterial species is present during all cases of BV by any definition. Therefore, no single bacterium could be considered a specific marker for the diagnosis of BV, and the interaction between microorganisms acting in consort in the human vaginal environment needs to be considered [47]. Among the Firmicutes, *Streptococcus* and *Staphylococcus* genera were detected, which could be associated with BV, and the genus *Lactobacillus* represented only about 17% of the BV vaginal bacterial biota, dominated by *L. fermentum*, *L. casei*, *L. jonsonii*, and *L. salivarius* species. It is noteworthy that vaginal *Lactobacillus* species can create a barrier against invasion by pathogens, since the products of their metabolism secreted in the cervicovaginal fluid play an important role in the inhibition of bacterial and viral infections [48]. In addition, the low vaginal pH (< 4.5) caused by the production of lactic acid by members of the genus *Lactobacillus* tends to suppress the growth of the pathogenic microorganisms that are mainly responsible for vaginal dysbiosis. The vaginal pH is the key factor in the increased incidence of BV in the reproductive age group, and many adjuvant drugs, such as ascorbic acid, *Lactobacillus* strains, and probiotics, have been investigated to try to decrease vaginal pH and thus reduce the recurrence of BV [49–51]. However, until the pathogenesis of BV is completely understood, treatment will remain unsatisfactory. Clinicians use various regimens for treating BV and a current treatment strategy includes the administration of antibiotics such as metronidazole or clindamycin, either orally or topically [52,53]. Although many women respond to antibiotics, BV recurs in 11–29% of women at 1 month [52,54,55] and an adherent *G*. *vaginalis* biofilm persists after the antibiotic therapy [56]. Bacteria in biofilms respond differently to antibiotic treatment, showing higher resistance, compared with their planktonic counterparts [57–60].

In the present study, a promising therapeutic approach based on topical lactoferrin administration for the treatment of BV, was proposed. Lactoferrin, at two different concentrations (100 mg and 200 mg), was administered to women with BV and its ability to modify the vaginal bacterial biota, both during and after administration, was studied in depth. The results demonstrated that both concentrations significantly increased the level of lactobacilli and decreased pathogenic bacteria such as *Gardnerella*, *Prevotella*, and *Lachnospira* during the treatment, in accordance with previous studies [31,33]. Differences were detected 2 weeks after administration. While in the BV patients treated with 200 mg of lactoferrin the lactobacillus population continued to increase, inhibiting the growth of pathogens, in the BV patients treated with 100 mg of lactoferrin a slight decrease in lactobacilli and a concomitant increase in *Gardnerella* and *Prevotella* genera were registered. Based on our results, among the genus *Lactobacillus*, *L. helveticus* was one of the dominant species detected during both lactoferrin 100 mg and 200 mg treatments. *Lactobacillus helveticus* is a homofermentative, Gram-positive, rod-shaped thermophilic microorganism belonging to the lactic acid bacteria, generally used in the dairy industry as a starter culture in the manufacture of several Italian cheeses. Several studies have demonstrated that *L. helveticus* exhibits health-promoting properties [61], stimulating the immune system, increasing defense against pathogens, and influencing the intestinal microbiota composition [62,63]. It is a resilient microorganism of the human gastrointestinal tract and is considered a transient species of fecal origin in the vagina [64]. The beneficial effects exerted by *L. helveticus* in this environment are of particular relevance to recurrent vulvovaginal candidiasis, mainly caused by *C*. *albicans* strains that can originate from prolonged antimicrobial treatments [65]. In addition, it was demonstrated *in vitro* that *L. helveticus* interfered with the adhesion of pathogens on the urovaginal surface, reducing the viability of vaginosis-associated bacteria *G*. *vaginalis* and *P. bivia*, and increasing the Lactobacillaceae [66]. Further studies are needed to investigate the health-promoting properties of *L. helveticus* isolates from the vaginal ecosystem, to elucidate the characteristics that allow them to successfully colonize the vagina, and to discover the key factors enhancing the selection of specific microorganisms.

## Conclusion

Our data indicated that the women with BV enrolled in the present study were colonized by more than a single species of *Lactobacillus*, which significantly increased during and after treatment with lactoferrin. *Lactobacillus helveticus*, not previously detected in the vaginal ecosystem, was the most abundant species found after lactoferrin treatment, especially at 200 mg. The results clearly highlight the beneficial effects of lactoferrin as a promising therapeutic approach for BV.

## Supplementary Material

Supplementary material
