# Supplementary material for: Bacterial biota of women with bacterial vaginosis treated with lactoferrin: an open prospective randomized trial
Source: Microb Ecol Health Dis. 2017 Jan 1;28(1):1357417. doi: 10.1080/16512235.2017.1357417 (PMC5614382; doi:10.1080/16512235.2017.1357417)
Supplement: Supplementary material [file ZMEH_A_1357417_SM6444.zip › supplementary tables and figure legend.docx]

**Supplementary tables and figure legend**

TABLE S1. Demographic data, sign and symptoms of BV patients of A and B groups at baseline.

TABLE S2. Number of sequences analyzed, biodiversity measures and estimated sample coverage (%) of total 16S rRNA gene of vaginal samples.

Figure S1. Rarefaction curves of A and B group samples.
