# Supplementary material for: Bacterial biota of women with bacterial vaginosis treated with lactoferrin: an open prospective randomized trial
Source: Microb Ecol Health Dis. 2017 Jan 1;28(1):1357417. doi: 10.1080/16512235.2017.1357417 (PMC5614382; doi:10.1080/16512235.2017.1357417)
Supplement: Supplementary material [file ZMEH_A_1357417_SM6444.zip › Table S1 rev.docx]

| Variable | Group A (n=28)* | Group B (n=30)* |
| --- | --- | --- |
| Age (years) (mean ± SD) | 30.14 ± 10.2 | 31.41 ± 9.6 |
| Body weight (Kg) (mean ± SD) | 58.3 ± 8.6 | 60.1 ± 9.0 |
| Height (cm) (mean ± SD) | 167 ± 5.3 | 165 ± 6.1 |
| Itching ** | 28 (100) | 30 (100) |
| Burning ** | 26 (92.8) | 26 (86.7) |
| Dysuria ** | 26 (92.8) | 27 (90.0) |
| Odour ** | 28 (100) | 30 (100) |

*Number of patients that completed the study

**Results are expressed as absolute number of patients that showed these symptoms with percentage in parenthesis
