# Supplementary material for: Bacterial biota of women with bacterial vaginosis treated with lactoferrin: an open prospective randomized trial
Source: Microb Ecol Health Dis. 2017 Jan 1;28(1):1357417. doi: 10.1080/16512235.2017.1357417 (PMC5614382; doi:10.1080/16512235.2017.1357417)
Supplement: Supplementary material [file ZMEH_A_1357417_SM6444.zip › Table S2 rev.docx]

|  | | **A** | | | | **B** | | |
| --- | --- | --- | --- | --- | --- | --- | --- | --- |
|  |  | T0 | T1 | T2 | T0 | | T1 | T2 |
| N° reads | 95862 | | 53256 | 41246 | 71491 | | 50486 | 55972 |
| OTUs | 80 | | 71 | 37 | 48 | | 35 | 70 |
| Chao 1 | 252 | | 96 | 62 | 239 | | 155 | 210 |
| Shannon index | 3.148 | | 2.309 | 0.103 | 2.219 | | 0.990 | 1.612 |
| Good’s coverage (%) | 99.4 | | 99.4 | 99.1 | 99.2 | | 99.6 | 99.5 |
