# Supplementary figures and images for: Bacterial biota of women with bacterial vaginosis treated with lactoferrin: an open prospective randomized trial
Source: Microb Ecol Health Dis. 2017 Jan 1;28(1):1357417. doi: 10.1080/16512235.2017.1357417 (PMC5614382; doi:10.1080/16512235.2017.1357417)

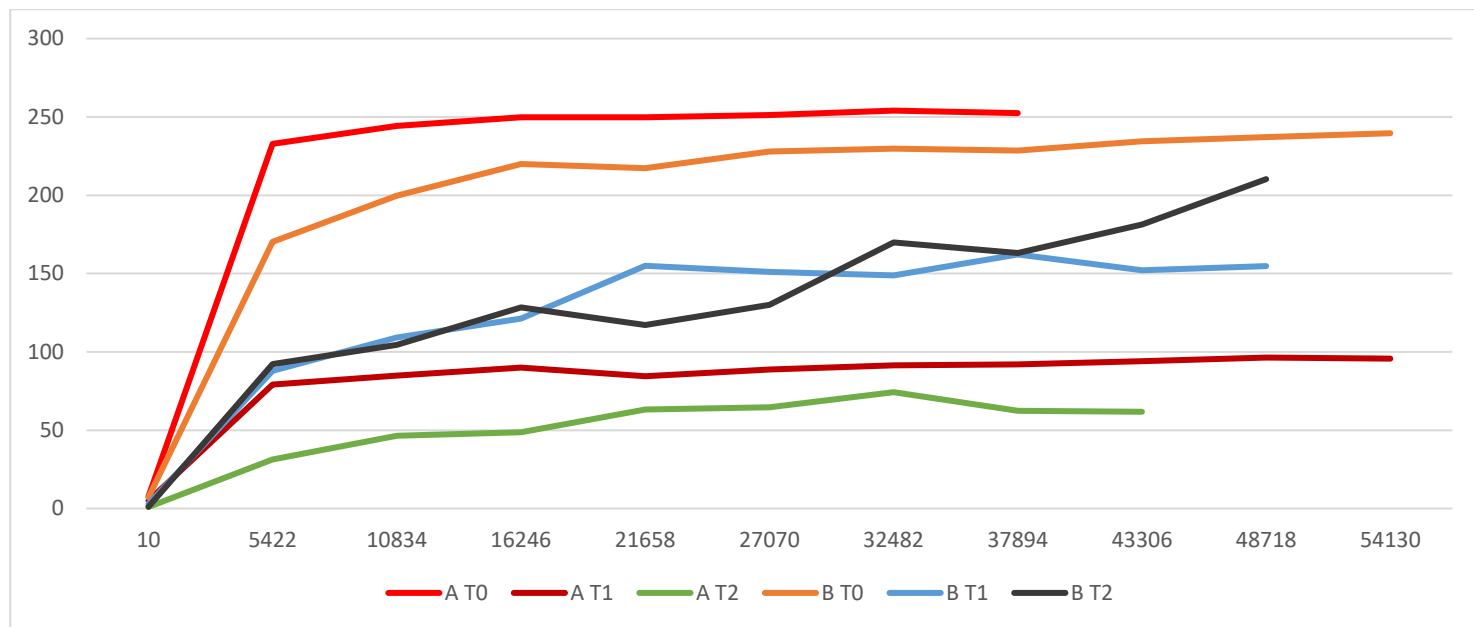

Supplement: Supplementary material [file ZMEH_A_1357417_SM6444.zip › Fig 1S.pdf]
